# P-1213. Optimizing the synergy between Aztreonam & Avibactam for NDM plus OXA48 producing Carbapenem resistant Klebsiella pneumoniae infections: an innovative infusion method

**DOI:** 10.1093/ofid/ofae631.1395

**Published:** 2025-01-29

**Authors:** Rajeev Soman, Aashna Gandhi, Noopur Kedare, Geethu Joe, Saiprasad Patil, Amullya Pednekar, Hanmant Barkate

**Affiliations:** Jupiter Hospital, Pune, Pune, Maharashtra, India; Jupiter Hospital, Pune, Pune, Maharashtra, India; Jupiter Hospital, Pune, Maharashtra, India; Jupiter Hospital, Pune, Pune, Maharashtra, India; Glenmark Pharmaceuticals Limited, Mumbai, Maharashtra, India; Glenmark Pharmaceuticals Limited, Mumbai, Maharashtra, India; Glenmark Pharmaceuticals Limited, Mumbai, Maharashtra, India

## Abstract

**Background:**

Ceftazidime-avibactam (CAZ AVI) along with aztreonam (ATM) is used off-label for NDM+OXA48 producing carbapenem resistant Klebsiella pneumoniae (CR Kp). If the level of AVI is maintained at 2.5 μg/ml, it protects ATM from OXA48 & allows it to act against such CR Kp. In such a situation the MIC to ATM has been found in a published report to be 2 μg/ml.

**Figure:**
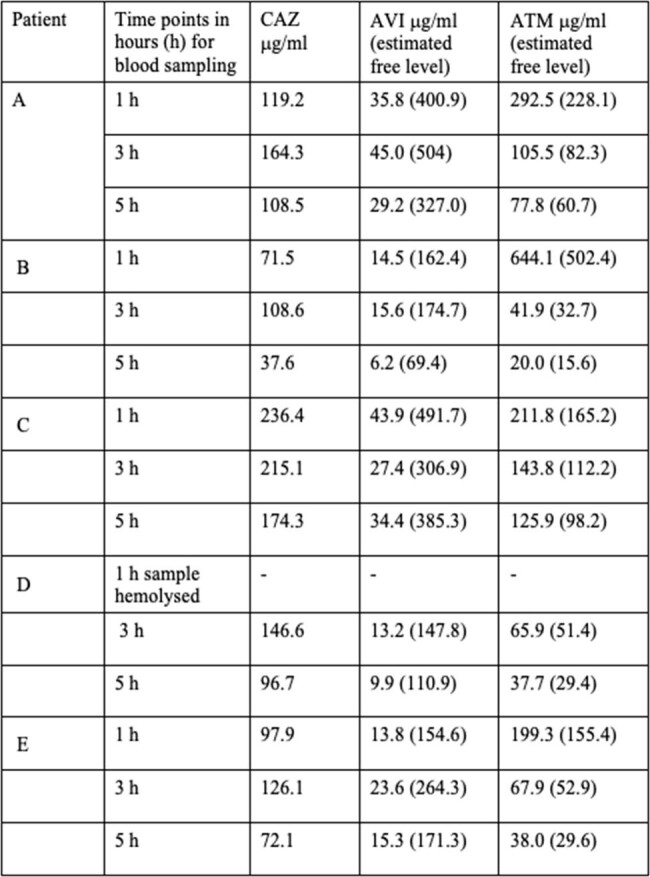

Table shows the measured protein bound drug levels. Estimated free drug levels are shown in brackets.

**Methods:**

We used CAZ AVI (CAZ 2 grams and AVI 0.5 gram) infusion over 3 hours & ATM 2 grams infusion over the 2nd half an hour as shown in figure 1, in 5 patients infected with NDM+OXA48 CR Kp, as a proof-of-concept PK study. The rationale of starting the ATM infusion after the CAZ AVI infusion is begun is to allow AVI levels to build up & prevent inactivation of ATM by ESBL & OXA48. The administration of ATM over half an hour, rather than by a more prolonged infusion, may ensure a relatively high initial level of ATM, which is expected to remain for a considerable time owing to its long half life.

**Figure:**
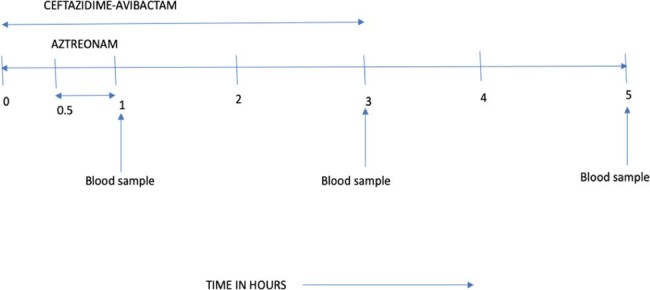

Infusion strategy for Ceftazidime-Avibactam (CAZ-AVI) & Aztreonam (ATM)

**Results:**

After measurement of protein bound levels, free AVI (*f*AVI) & free ATM (*f*ATM) levels were estimated from the known extent of protein binding. As shown in table 1, all levels of estimated *f*AVI were above 2.5 mcg/ml. The estimated levels of *f*ATM, at 5 hours after beginning the infusion were at or above 15.6 mcg/ml, with a mean of 45.6 mcg/ml & range of 15.6 to 98.2 mcg/ml. Hence time above 4 times the assumed ATM MIC of 2 mcg/ml, for more than 50% of the dosing interval was easily achieved in all the patients.

**Conclusion:**

Thus, the PK/PD index obtained is higher than that needed for optimal killing & can suppress resistance which may be useful from the perspective of antimicrobial stewardship.

**Disclosures:**

**Rajeev Soman, MBBS, MD (General Medicine), FIDSA**, Astellas: Honoraria|Biomeriux: Honoraria|Cipla: Honoraria|Emcure: Honoraria|Gilead: Honoraria|Glenmark: Honoraria|Gufic: Honoraria|Intas: Honoraria|MSD: Honoraria|Mylan: Honoraria|Pfizer: Honoraria **Saiprasad Patil, Global Medical Affairs**, Glenmark Pharmaceuticals Limited: Employee **Amullya Pednekar, Global Medical Affairs**, Glenmark Pharmaceuticals Limited: Employee **Hanmant Barkate, Global Medical Affairs**, Glenmark Pharmaceuticals Limited: Employee

